# Itaconic acid degradation in *Aspergillus niger*: the role of unexpected bioconversion pathways

**DOI:** 10.1186/s40694-018-0062-5

**Published:** 2019-01-04

**Authors:** Abeer H. Hossain, Alexander Ter Beek, Peter J. Punt

**Affiliations:** 1 Dutch DNA Biotech B.V, Padualaan 8, 3584 CH Utrecht, The Netherlands; 2 Molecular Biology and Microbial Food Safety, University of Amsterdam, Science Park 904, 1098 XH Amsterdam, The Netherlands

## Abstract

**Background:**

Itaconic acid (IA), a C5-dicarboxylic acid, has previously been identified as one of the top twelve biochemicals that can be produced by biotechnological means. IA is naturally produced by *Aspergillus terreus*, however, heterologous production in the related species *Aspergillus niger* has been proposed earlier. Remarkably, we observed that during high producing conditions and elevated titers *A*. *niger* detoxifies the extracellular medium of IA. In order to determine the genes responsible for this decline in IA titers a transcriptome analysis was performed.

**Results:**

Transcriptome analysis has led to the identification of two novel and previously unknown IA bioconversion pathways in *A*. *niger*. One pathway is proposed to convert IA into pyruvate and acetyl-CoA through the action of itaconyl-CoA transferase (IctA), itaconyl-CoA hydratase (IchA) and citramalyl-CoA lyase, similar to the pathway identified in *A*. *terreus*. Another pathway putatively converts IA into 1-methyl itaconate through the action of trans-aconitate methyltransferase (TmtA). Upon deleting the key genes *ictA* and *ichA* we have observed increased IA production and titers and cessation of IA bioconversion. Surprisingly, deletion of *tmtA* lead to strong reduction of heterologous IA production.

**Conclusion:**

Heterologous IA production in *A*. *niger* induces the expression of IA bioconversion pathways. These pathways can be inhibited by deleting the key genes *ictA*, *ichA* and *tmtA*. Deletion of *ictA* and *ichA* resulted in increased IA production. Deletion of *tmtA*, however, resulted in almost complete cessation of IA production.

**Electronic supplementary material:**

The online version of this article (10.1186/s40694-018-0062-5) contains supplementary material, which is available to authorized users.

## Background

Rising carbon emissions due to increased industrialization and its effect on the climate are raising awareness to organize our economy in more sustainable ways. However, to transition from our current fossil resource-based economy to a bio-based economy is not easily achieved given the huge dependency on fossil fuels for energy and commodity needs. Biotechnologically produced organic acids have great potential as sustainable alternative for petrochemicals and its use as commodities [1]. The main bottleneck for industrial application of biochemicals, however, is the high price compared with petrochemicals. Many yeasts and bacteria have been exploited in the production of industrially relevant biochemicals, e.g. succinic acid [2]. However, filamentous fungi are due to their natural lifestyle as saprophytic organisms well equipped to break down complex carbohydrate structures e.g. lignocellulose and produce industrially relevant biochemicals. Apart from this fact filamentous fungi are also known as efficient organic acid producers, in particular members of the genus *Aspergillus* [3].

Itaconic acid (IA), a C5-dicarboxylic acid, has previously been identified as one of the top twelve biochemicals that can be produced by biotechnological means [4]. The potential applications of IA in green chemistry are numerous and an exciting overview of novel applications is provided by Robert and Friebel [5]. Industrial production of IA is performed using the natural producer *A*. *terreus*. Currently IA and its chemical derivatives are seen as niche chemical with low industrial relevance. The main applications of IA and its chemical derivatives are as superabsorbent polymers, synthetic latex, detergent builders, polymers and polyester resins [6, 7]. Lowering production costs and selling price of IA could result in the promotion of IA into a platform chemical that would lead to an tenfold increase in its market size and open market applications such as thermoplastics [8, 9]. In order to achieve this feat the selling price of IA should be competitive with fossil-based end use chemicals such as maleic anhydride. The high production cost and selling price of IA can be attributed to the sensitivity of *A*. *terreus* to impurities in industrial cultivation medium and tight control of fermentation processes [10–12].

Heterologous IA production in the related species *Aspergillus niger* has been proposed earlier based on the superior organic acid production capabilities of *A*. *niger* as exemplified by industrial citric acid (CA) production that mainly employs *A*. *niger* [13–15]. Additionally *A*. *niger* is a more robust production organism able to withstand impurities in industrial cultivation medium; a feat that is evident by the low selling prices of biotechnologically produced CA [16, 17]. In our previous report we have communicated the rewiring of *A*. *niger* secondary metabolism citrate synthase (CitB) leading to an increased IA yield, titer and productivity [18]. Metabolic engineering of ATP-citrate lyase in our rewired pathway has resulted in further improvement of IA production (Hossain AH et al. manuscript in preparation). However, we also observed in cultivations with these engineered strains that IA titer reaches a plateau after which titers start to decline rapidly (Hossain AH et al. manuscript in preparation). This decline in IA is supposedly brought about by putative IA bioconversion. In order to determine the genes responsible for IA bioconversion a transcriptome analysis was performed. In combination with a previous transcriptome analysis of primary metabolism genes related to glycolysis, TCA cycle and organic acid transport (de Vries et al. [3]), this analysis led to the identification of previously unknown genes of which the expression is highly upregulated in IA producing conditions.

## Materials and methods

### Strains and media components

*Aspergillus niger* strain CitB#99 (CBS141659) [18] was used in this study in which deletion of *ictA*, *ichA* and *tmtA* was performed. The strains used for transcriptome analysis are listed in Table 1. All strains were stored in 30% glycerol at − 80 °C and maintained on agar containing minimal medium (MM) plates (16 g/L agar, 6 g/L NaNO_3_, 0.52 g/L KCl, 1.52 g/L KH_2_PO_4_, 10 g/L glucose, 0.0022 g/L ZnSO_4_.7H_2_O, 0.0011 g/L H_3_BO_3_, 0.0005 g/L MnCl_2_.4H_2_O, 0.0005 g/L FeSO_4_.7H_2_O, 0.00017 g/L CoCl_2_.6H_2_O, 0.00016 g/L CuSO_4_.5H_2_O, 0.00015 g/L NaMoO_4_.2H_2_O, 0.005 g/L Na_2_EDTA and 0.5 g/L Mg_2_SO_4_). Spore suspensions were prepared using physiological salt solution (0.9% NaCl) and stored at 4 °C for up to 1 year. Fresh spore suspensions were prepared for inoculation during each shake flask and batch fermentation experiment.

**Table 1:** List of strains used for transcriptome analysis

Strain	Abbreviation	Strain description
AB1.13 *pyrG*+	AB1.13	Uridine prototroph of AB1.13 *pyrG*—[19]
AB1.13 CAD 4.1	AB1.13 CAD	Selected pyrG + transformant of *cadA* expressing transformant (CAD10.1) of AB1.13 [20]
AB1.13 CAD + MTT + MFS_3	AB1.13 CAD + MTT + MFS	Selected *mfsA* expressing transformant of MTT 1.4 [19]
AB1.13 CAD + MFS + MTT #49B;	AB1.13 #49B	Selected *mttA* expressing transformants of AB1.13 CAD + MFS 3.9 [18]
AB1.13 CAD + MFS + MTT + CitB #99; #113	CitB#99; CitB#113	Selected *citB* overexpressing transformants of AB1.13 CAD + MFS + MTT #49B [18]

### Split marker deletion construct and transformation

Auxotrophic *pyrE*-strains were generated by cultivating CitB#99 on 5-fluoroorotic acid selective plates [21]. Plates were incubated in a 33 °C stove for 3–5 days until colony formation was visible. Organic acid production of CitB#99 *pyrE*-colonies was tested by cultivation in microtiter plates (see section screening). Knock-out of *ictA*, *ichA* and *tmtA* was performed using the split-marker method [22]. An schematic overview of the split-marker approach is shown in Additional file 1. Split-marker flanks for an *ictA* and *ichA* knock-out were generated with fusion PCR and split-marker flanks for a *tmtA* knock-out were in vitro synthesized by GeneArt Gene Synthesis (Thermo Scientific) and individually introduced into pJET1.2/blunt via blunt-end ligation using the CloneJET PCR Cloning Kit (Thermo Scientific). The primers used to create the split-marker fragments for *ictA* and *ichA* are listed in Additional file 2. The *pyrE* gene of *Aspergillus oryzae* RIB40 (AO090026000521) was employed to complement the *pyrE* deficient phenotype. The split-marker fragments were co-transformed in an ratio of 1:1 (5 µg flank 5′: 5 µg flank 3′). All transformations were carried out according to the protocol as reported by Punt et al. [23]. Transformed protoplasts were plated on MM agar plates containing sorbitol and grown at 33 °C for 3–5 days until colonies were visible. Successful targeted integration of bi-partite fragments were determined with diagnostic colony PCR [24] using various primer combinations. Colonies that showed the expected PCR fragments were selected for further screening in microtiter plate and shake flask experiments.

### Microtiter plate transformant screening

Plates carrying transformed cells were allowed to grow and sporulate for 1–2 weeks after which individual colonies were transferred to a selective MM plate. Individual colonies from this plate were each streaked on a separate selective MM plate to isolate single colonies that in turn were used to inoculate 1 mL liquid cultures in a 96-wells deepwell plate (Axygen; Corning, NY) containing M12 ++ medium (1.43 g/L NH_4_NO_3_, 0.11 g/L KH_2_PO_4_, 0.5 g/L MgSO_4_ × 7 H_2_O, 0.005 g/L CuSO_4_ × 5 H_2_O, 0.0006 g/L FeIIICl_3_ × 6 H_2_O, 0.0006 g/L ZnSO_4_ × 7 H_2_O, 0.074 g/L NaCl, 0.13 g/L CaCl_2_ × 2 H_2_O and 100 g/L glucose) [20]. This 96-wells plate was incubated for 72 h at 33 °C and 850 RPM. Supernatant was filtered over a 0.22 µM filter (Corning; Corning, NY) and analyzed on an HPLC for IA production (see below).

### Flask cultivations

MM agar plates were streaked with conidia from glycerol stocks or from isolated single colonies that were determined by colony PCR. These plates were incubated at 33 °C for several days till plates were fully grown. Fresh conidia suspensions were prepared by harvesting conidia from these plates with sterile 0.9% NaCl solution. The harvested conidia were counted on the LUNA II cell counter (Logos Biosystems). Non-baffled shake flasks (500 mL) were filled with 100 mL M12 ++ medium and inoculated with 1.0 × 10^6^/mL conidia and incubated at 35 °C and 0 RPM. Flasks were weighed when empty, after inoculation and each day before sampling. Evaporation is calculated from the measured weight of the flasks and used to correct measured concentrations of organic acids and glucose by HPLC (see below). Error bars in graphs of flask cultivations indicate the standard error of the mean. All flask cultivations were performed in duplicate.

### Controlled-batch cultivations

Controlled-batch cultivations were performed on 5L scale benchtop New Brunswick Scientific fermenters (BioFlo 3000) at 33 °C. Starting pH was 3.5 after inoculation and medium was allowed to naturally acidify till pH 2.3 and then kept at pH 2.3 by addition of 4 M KOH. Dissolved oxygen (DO) tension was 25% at the moment of inoculation and when DO dropped till 20% it was kept at 20%. The system was calibrated with 100% sterile air as 100% DO and 100% N_2_ as 0% DO. The fermenter was inoculated by 72 h old 100 mL non-baffled shake flask cultures containing 1.0 × 10^8^ spores. Medium composition for fermentation and pre-culture (M12 ++) is described above.

### HPLC

Metabolite analysis was performed using a WATERS e2695 Separations Module equipped with an Aminex HPX-87H column (Bio-Rad) and 5 mM H_2_SO_4_ as eluent. Detection of peaks occurred simultaneously by a refractive index detector (WATERS 2414) and a dual-wavelength detector (WATERS UV/Vis 2489). Data processing was done with Empower Pro software (Empower 2 Software, copyright 2005–2008, Waters Corporation, Milford, MA, USA).

### RNA isolation and transcriptome analyses

Biomass samples for RNA isolation were taken at several time points during fermentation and washed with distilled water and frozen in liquid N_2_. The mycelium was disrupted by bead-beating with 0.1 mm acid-washed Zirconium-Silica beads and RNA extraction proceeded using the ChargeSwitch RNA extraction protocol from Invitrogen (Carlsbad, CA, USA). Quality control was checked on 1 × 3-(*N*-morpholino)propanesulfonic acid/6% Formaldehyde agarose gels and stained with ethidium bromide.

BaseClear in Leiden, NL performed digital gene expression profiling experiments based on RNA-Seq with an Illumina HiSeq 2000 System. Approximately 8–32 M unfiltered paired-end (PE) reads (99 bp/read on ~ 320 bp cDNA inserts) were obtained. Reads were trimmed of the first 2 bases of the 5′ end because these bases showed an aberrantly low GC content. The reads were then further filtered, such that all quality Phred scores after filtering are at least 22, with a read-length of at least 40 bases. Around 70–80% of the bases passed these criteria (including a 2% loss because of clipping). After filtering the # PE-reads/samples were between 7.6 and 19.8 M for all the samples respectively.

Reads were aligned to the 20 contigs in a FastA file of the *Aspergillus niger* CBS 513.88 reference genome (from http://www.ebi.ac.uk/ena). Source EMBL annotations were converted to GFF format. The embl data appeared to be derived from multiple sources with different feature tags. These were converted to one uniform GFF format that could be accepted by our third-party software (consistent gene_ids across all contigs). Missing gene definitions (e.g. inserted genes for IA production) were inserted. The reads were aligned to the reference genome using software based on a Burrows–Wheeler Transform (BWT) algorithm. A mismatch rate of 4% was allowed for the alignment. The maximum insertion length was 3. The maximum deletion length was 3. All samples had more than 85% of the reads aligned, resulting in SAM alignment files. Gene expression was measured as the number of aligned reads to reference genes and was normalized to RPKM values (Reads Per Kb per Million reads; Mortazavi et al. [25]). Hierarchical clustering was performed with TIGR MEV 4.0. A stringent cut-off at 2logR value of 4.0 for upregulated genes and − 4.0 for downregulated genes was held for data analysis. A more relaxed cutoff of > 2.0 or smaller − 2.0 was used to explore the data for identifying novel differentially expressed gene clusters.

## Results

### Transcriptome analysis of high IA producing *A*. niger strains

Previously we have reported the IA production of *A*. *niger* strain CitB#99 that reaches a final titer of 26.2 g/L with max productivity of 0.35 g/L/h and yield of 0.37 g/g [18]. IA production was further improved by metabolic engineering of ATP-citrate lyase (Hossain et al. [18]). Remarkably, we have also observed IA bioconversion in *A*. *niger* during IA producing cultivations. This observation manifested in strongly reduced IA titers after achieving a peak IA titer. In addition, IA bioconversion was also observed in cultures were exogenous IA was added to shake flask cultures, showing a reduction of IA levels (Additional file 3). In order to identify the genes involved in IA bioconversion we have analyzed a transcriptome dataset of biomass isolated from batch fermentations with low, medium and high IA producing *A*. *niger* strains. In Table 2 transcriptome results of differentially regulated genes between the high IA producing strain CitB#99 and AB1.13 WT, that does not produce IA, is given. As expected, the four genes that constitute the engineered part of the heterologous IA biosynthesis pathway i.e. *cadA*, *mfsA*, *mttA* and *citB* are all highly expressed in CitB#99 (Table 2A).

**Table 2:** Differential expressed genes in high IA producing strains

A				2logR values				RPKM values			
Locus tag	Old locus tag	Annotation	TargetP	AB1.13	AB1.13 CAD	AB1.13 #49B	CitB#99	AB1.13	AB1.13 CAD	AB1.13 #49B	CitB#99
ANI_1_1474074	An08g10920	Citrate synthase (CitB)	Cytosol	0.02	0.04	− 0.05	11.40	3.05	3.10	2.87	10,838.65
*cadA* (*A*. *terreus*)		Cis-aconitate decarboxylase	Cytosol	0.05	10.62	9.47	10.72	3.54	6887.96	3116.06	7417.64
ANI_1_1906104	An12g05750	MFS phosphate transporter	PM	0.13	1.02	10.28	10.55	1.09	2.87	2375.80	2852.93
*hph* (S. hindustanus)		Hygromycin B 4-*O*-kinase	Cytosol	0.23	0.17	8.84	10.04	0.94	0.86	759.61	1743.36
*mttA* (*A*. *terreus*)		Putative mitochondrial tricarboxylite transporter	Mito	− 0.18	0.18	8.46	9.86	0.19	0.54	477.02	1257.78
*mfsA* (*A*. *terreus*)		Putative major facilitator superfamily transporter A	PM	0.07	1.44	7.49	9.19	0.30	2.36	222.56	721.88
*amdS* (A. nidulans)		Acetamidase A	Cytosol	0.00	8.57	7.57	7.90	0.37	517.76	259.47	325.00
ANI_1_2022014	An01g14940	Phospholipase C PLC-C	Secreted	0.06	0.08	4.99	7.18	8.40	8.53	286.39	1310.11
ANI_1_1486074	An08g11030	3-Phytase B	Secreted	− 0.03	0.21	5.94	6.99	27.36	32.28	1771.34	3663.34
ANI_1_2368024	An02g02840	Sialidase	Secreted	− 0.05	0.06	7.22	6.81	0.21	0.30	184.78	139.52
ANI_1_236084	An09g02180	Lipase	Secreted/Mito	− 0.34	− 0.30	3.96	6.78	2.43	2.55	67.01	479.51
ANI_1_1848144	An16g06190	Metabolite transport protein GIT1	PM	0.04	0.73	6.80	6.46	6.81	11.62	846.83	669.87
ANI_1_166144	An16g01340	Glyoxalase domain-containing protein 5	Mito/Cytosol	0.24	4.70	5.70	6.20	16.28	379.47	761.69	1080.98
ANI_1_248124	An14g01550	Acid phosphatase	Secreted	− 0.16	− 0.27	4.91	5.89	4.20	3.82	174.16	345.29
ANI_1_1500104	An12g02320	Short-chain dehydrogenase/reductase	Cytosol	0.01	3.07	4.70	5.65	2.33	26.63	84.56	164.88
ANI_1_1330084	An09g03700	Hypothetical protein	Secreted	− 0.14	0.10	4.05	5.64	0.46	0.72	25.72	79.39
ANI_1_460094	An11g03340	Alpha-amylase A type-1/2	Secreted	− 0.12	0.33	1.56	5.52	12.80	17.81	43.27	683.63
ANI_1_542164	An18g04140	Acid phosphatase	Secreted	0.04	0.07	4.04	5.50	1.42	1.45	37.62	104.94
ANI_1_1576144	An16g02440	Phytase-like	Secreted	− 0.03	0.04	4.27	5.48	3.98	4.23	97.10	226.06
ANI_1_260144	An16g01850	BYS1 domain protein	Secreted	− 0.64	− 0.16	2.35	5.30	9.22	13.19	79.91	623.78
ANI_1_1250124	An14g02660	NPP1 domain protein	Secreted	0.12	0.24	4.08	5.29	3.11	3.48	63.27	147.19
ANI_1_2836024	An02g08830	Histone transcription regulator 3	Nucleus	0.00	0.09	− 0.03	5.15	9.48	10.11	9.28	371.63
ANI_1_652114	An13g01750	Acid phosphatase	Secreted	0.06	0.10	4.67	5.13	70.73	72.83	1754.06	2422.06
ANI_1_1440124	An14g04660	Aromatic ring-opening dioxygenase, catalytic LigB subunit	Cytosol/Mito/Per	0.13	0.32	0.32	5.13	2.59	3.10	3.09	113.67
ANI_1_1432064	An07g00760	Putative itaconyl-CoA transferase (ictA)	Mito	0.32	3.27	3.92	5.06	13.07	108.35	170.34	375.89
ANI_1_1812184	An04g04240	Phosphate transporter	PM	0.27	1.05	4.83	4.99	2.94	5.74	91.74	102.55
ANI_1_126034	An03g01120	Aromatic-amino-acid aminotransferase	Nucleus	− 0.34	3.62	4.24	4.98	29.79	478.24	735.44	1226.92
ANI_1_246114	An13g01760	Oligopeptide transporter	PM	0.33	0.46	2.43	4.97	1.30	1.51	8.88	56.23
ANI_1_1908104	An12g05810	Multicopper oxidase	Secreted	0.04	0.20	4.33	4.76	0.18	0.31	21.98	30.01
ANI_1_2118064	An07g09220	Putative itaconyl-CoA hydratase (ichA)	Mito	− 0.11	2.78	4.04	4.71	7.32	60.28	146.61	232.65
ANI_1_1324074	An08g09850	Acid phosphatase	Secreted	− 0.18	− 0.36	3.70	4.58	128.06	112.96	1896.57	3490.63
ANI_1_1520134	An15g04760	Glycosyl hydrolase family 71 protein	Secreted	− 0.29	− 0.82	2.21	4.57	1.58	0.79	13.68	74.04
ANI_1_2122184	An04g08320	MFS monocarboxylate transporter	PM	0.17	0.49	1.25	4.46	7.11	9.16	16.14	158.52
ANI_1_3198024	An02g13080	Phenylacetate-coenzyme A ligase	Cytosol	− 0.28	− 0.51	0.35	4.28	0.27	0.08	0.96	28.87
ANI_1_1430064	An07g00750	Fungal transcription factor	Nucleus/Mito	0.02	3.04	3.73	4.28	0.52	11.37	19.01	28.19
ANI_1_820034	An03g06550	Glucoamylase	Secreted	0.13	− 0.02	1.76	4.18	243.81	218.86	756.84	4054.75
ANI_1_1020134	An15g07520	Phosphoglycerate mutase family protein	Cytosol	− 0.20	− 0.60	4.75	4.18	12.67	9.31	420.93	282.80
ANI_1_1810184	An04g04230	Phosphate transporter (reannotated)	Nucleus/Cytosol	− 0.18	0.33	4.60	4.17	4.67	7.06	154.52	114.01
ANI_1_294044	An05g02340	Extracellular dihydrogeodin oxidase/laccase	Secreted	− 0.08	− 0.07	4.48	4.11	0.44	0.44	32.96	25.14
ANI_1_864144	An16g06510	Trans-aconitate 2-methyltransferase (tmtA)	Cytosol	0.08	0.73	2.40	4.08	12.71	20.51	67.34	218.46
ANI_1_342114	An13g02590	Sugar transporter	PM	− 0.01	0.28	2.16	4.06	3.21	4.16	18.01	69.60
ANI_1_1098184	An04g06920	Alpha-glucosidase	Secreted	0.00	− 0.33	0.57	4.04	174.34	138.54	258.87	2884.10
ANI_1_1820094	An11g03450	Small secreted protein	Secreted	0.33	1.68	2.25	4.02	58.07	149.69	222.30	763.49
ANI_1_330024	An02g02480	MFS phosphate transporter	PM	− 0.20	1.65	3.18	4.02	3.12	13.82	41.86	75.60
ANI_1_762014	An01g05900	Cytochrome P450	Secreted	− 0.13	− 0.10	3.86	4.00	0.63	0.66	24.97	27.63
ANI_1_1778074	An08g02590	GPI anchored protein	Secreted	− 0.06	0.21	3.26	4.00	10.62	13.07	115.12	192.81

B				2logR values				RPKM values			
Locus tag	Old locus tag	Annotation	TargetP	AB1.13	AB1.13 CAD	AB1.13 #49B	CitB99	AB1.13	AB1.13 CAD	AB1.13 #49B	CitB99
ANI_1_542134	An15g03800	Hydrophobin	Secreted	0.06	− 0.55	− 2.13	− 8.78	981.57	641.93	213.11	1.14
ANI_1_1416134	An15g04010	Hypothetical protein	Nucleus	0.27	− 0.20	− 5.87	− 7.79	288.73	208.03	3.11	0.09
ANI_1_92174	An10g00820	Oxaloacetate acetylhydrolase	Cytosol	− 0.13	− 0.81	− 2.61	− 7.51	1803.48	1122.69	323.44	9.81
ANI_1_220174	An10g00800	Purine nucleoside permease	Secreted	− 0.13	− 0.33	− 2.72	− 6.47	375.32	326.25	61.40	3.64
ANI_1_1046034	An03g01450	Major Facilitator Superfamily	PM	− 0.20	− 0.83	− 2.49	− 6.29	73.83	47.18	14.31	0.10
ANI_1_1062034	An03g01590	Arginine permease	PM	0.13	0.41	− 2.09	− 6.08	243.24	295.39	51.56	2.30
ANI_1_964034	An03g00580	Cytochrome P450 monooxygenase	Mito	− 0.29	− 1.86	− 3.52	− 6.00	228.74	76.05	23.45	3.39
ANI_1_966034	An03g00590	Trichodiene synthase Tri5	Cytosol	− 0.31	− 2.27	− 4.02	− 5.87	94.11	23.35	6.27	1.01
ANI_1_170034	An03g01460	1-Aminocyclopropane-1-carboxylate oxidase	Cytosol	− 0.10	− 0.86	− 2.52	− 5.43	114.81	67.35	20.61	1.88
ANI_1_1914064	An07g06460	C-7 hydroxycephem methyltransferase	Cytosol	− 0.26	− 0.32	− 4.38	− 5.28	36.47	35.03	1.16	0.16
ANI_1_182034	An03g01540	FAD dependent oxidoreductase	Secreted/Cytosol	0.05	− 0.42	− 1.58	− 5.26	78.94	56.69	24.80	1.02
ANI_1_1770144	An16g05290	Purine-cytosine permease	PM	− 0.05	0.00	− 1.13	− 5.22	838.03	867.97	395.45	22.38
ANI_1_990084	An09g00660	OPT oligopeptide transporter protein	PM	0.01	0.10	− 0.27	− 5.09	279.04	298.75	230.29	7.20
ANI_1_2008094	An11g05190	Type 1 glutamine amidotransferase	Cytosol	− 0.02	0.24	− 1.25	− 5.06	70.06	84.12	29.32	1.16
ANI_1_1366064	An07g00170	Hypothetical protein	Secreted	0.37	0.34	− 1.87	− 5.04	56.14	54.96	11.10	0.35
ANI_1_1572014	An01g11640	Ammonium transporter	PM	0.22	0.03	− 1.36	− 5.02	222.82	195.30	74.16	4.91
ANI_1_1862104	An12g05440	Urea active transporter 1	PM	− 0.15	− 0.26	− 2.63	− 5.01	68.04	63.09	11.38	1.38
ANI_1_2238104	An12g09660	Phosphoribosyl transferase	Cytosol	0.64	0.85	− 1.49	− 4.94	126.25	145.43	27.94	1.66
ANI_1_1456124	An14g04760	Male sterility domain containing protein	Cytosol	0.26	− 0.49	− 2.51	− 4.91	258.07	152.77	36.93	6.18
ANI_1_708024	An02g05060	Hypothetical protein	Secreted	− 0.03	− 1.15	− 1.98	− 4.81	810.00	371.87	208.67	28.41
ANI_1_2764024	An02g08200	Short-chain dehydrogenase/reductase	Cytosol	1.05	2.54	− 0.64	− 4.80	308.27	867.28	94.68	4.34
ANI_1_2236104	An12g09640	Uracil-regulated protein 1	Cytosol	0.44	0.67	− 0.72	− 4.80	107.20	125.82	47.45	1.86
ANI_1_46014	An01g00370	Aspartic endopeptidase (AP1)	Nucleus	0.10	− 0.18	− 1.58	− 4.79	1351.68	1115.18	422.54	44.77
ANI_1_1054034	An03g01520	12-oxophytodienoate reductase	Mito	− 0.03	− 1.01	− 1.20	− 4.71	42.16	20.82	18.17	0.68
ANI_1_1850094	An11g03640	OPT oligopeptide transporter family	PM	− 0.11	− 0.52	− 0.49	− 4.71	104.23	78.34	79.93	3.35
ANI_1_1990074	An08g05320	Short-chain dehydrogenase/reductase	Cytosol	− 1.43	− 1.61	− 3.15	− 4.70	16.50	14.51	4.33	0.82
ANI_1_474154	An17g01540	GABA permease	PM	0.07	0.26	− 2.34	− 4.67	85.82	98.28	15.40	2.26
ANI_1_1748104	An12g04260	NAD dependent epimerase/dehydratase	Mito	1.23	2.79	− 2.00	− 4.66	69.28	206.65	6.50	0.19
ANI_1_726064	An07g05830	Formamidase	Cytosol	− 0.03	0.02	− 0.57	− 4.58	267.27	276.94	184.40	10.45
ANI_1_3344014	An01g14400	Cercosporin toxin resistance protein CRG1	Cytosol	0.04	0.02	− 2.87	− 4.56	63.05	62.21	7.56	1.65
ANI_1_176044	An05g01410	Acyl-CoA oxidase	Mito	− 0.30	− 0.96	− 2.25	− 4.55	178.03	111.60	45.07	8.38
ANI_1_568054	An06g02150	DUF3328 protein	Secreted	0.11	0.34	− 4.28	− 4.55	48.49	57.10	1.36	0.96
ANI_1_1554124	An14g05980	MFS transporter	PM	0.01	0.48	− 3.43	− 4.54	29.12	40.79	1.78	0.29
ANI_1_1048034	An03g01470	d-galactonate transporter	PM	− 0.07	− 1.24	− 2.35	− 4.52	37.53	16.20	6.96	0.76
ANI_1_806074	An08g05670	Nitrate transporter	PM	0.13	1.08	− 1.66	− 4.44	44.15	86.17	12.00	0.90
ANI_1_3376014		Fe(II)/2-oxoglutarate (2OG) oxygenase	Cytosol	− 0.17	− 0.07	− 1.01	− 4.38	97.28	104.78	54.14	4.31
ANI_1_1340084	An09g03750	MAC/Perforin domain	Cytosol	0.10	0.11	− 2.61	− 4.37	22.58	22.73	2.58	0.06
ANI_1_1514034	An03g06020	4-Carboxymuconolactone decarboxylase	Cytosol	0.19	− 0.43	− 1.29	− 4.31	5568.62	3603.74	1987.79	245.12
ANI_1_2954014	An01g09970	Surface layer protein	Cytosol	0.37	− 0.03	− 1.62	− 4.29	66.37	50.05	16.01	1.67
ANI_1_2450014	An01g03790	Urea active transporter 1	PM	− 0.05	− 0.17	− 0.10	− 4.29	116.82	107.59	113.28	5.27
ANI_1_616114	An13g01470	Laminin gamma 3	Secreted	− 0.15	− 1.01	− 2.79	− 4.27	110.93	60.59	16.95	5.42
ANI_1_1456104	An12g01910	Phytase	Secreted	− 0.04	− 0.95	− 4.39	− 4.26	30.69	15.78	0.55	0.70
ANI_1_142174	An10g00180	Cupin_like	Cytosol/Mito	0.34	− 0.10	− 1.24	− 4.24	424.35	310.92	140.90	16.72
ANI_1_1362014	An01g09980	Asp-hemolysin	Secreted	0.32	− 0.14	− 1.07	− 4.21	347.94	252.51	132.21	14.06
ANI_1_878114	An13g03990	Nucleobase-cation-symport-1 (NCS1) transporter	PM	− 0.08	0.15	− 2.27	− 4.21	30.66	36.14	5.91	0.81
ANI_1_2456094		*S*-adenosylmethionine-dependent methyltransferase		0.08	− 0.27	− 3.01	− 4.20	69.27	54.39	7.29	2.63
ANI_1_2086074	An08g07010	DUF3445 protein	Mito	0.00	− 0.25	− 2.44	− 4.17	37.07	31.01	6.01	1.11
ANI_1_212174		Nitrilase		− 0.38	− 0.59	− 2.64	− 4.12	18.92	16.23	3.15	0.49
ANI_1_1828184	An04g04370	Phenylalanine ammonia-lyase	PM	0.17	− 0.10	− 2.11	− 4.11	277.24	229.84	56.44	13.37
ANI_1_76154	An17g00640	Zinc-binding alcohol dehydrogenase domain-containing protein cipB	Cytosol	1.04	2.86	− 2.13	− 4.11	119.10	425.44	12.36	2.40
ANI_1_1094134	An15g00280	Polysaccharide deacetylase	Cytosol	0.00	− 0.07	− 0.98	− 4.08	204.72	194.38	102.72	11.10
ANI_1_1144084	An09g01920	FAD binding domain protein	Secreted	0.75	2.11	− 1.49	− 4.04	39.96	103.97	7.67	0.48
ANI_1_294064	An07g02310	Glutathione *S*-transferase Ure2-like protein	Cytosol	0.01	− 0.86	− 2.83	− 4.02	187.81	102.94	25.42	10.62

(A) Genes upregulated in high IA producing conditions. Cutoff of 2logR values of 4.00 or higher was held. (B) Genes downregulated in high IA producing conditions. Cutoff of 2logR values of − 4.00 was held

Interestingly, among the highly upregulated genes are a large number of genes that encode gene products with domains that have functions in phosphate liberation and translocation. This result is in line with the fact that in the IA production medium phosphate is limiting [26]. Remarkably, also the expression of major secreted enzymes such as amylase and glucoamylase, but also a number of other secreted proteins is upregulated in high IA producing conditions. The mechanism behind this result is not entirely clear, but may also be related to the fact that in high IA secretion conditions stress responses are induced which may lead to improved protein secretion (Table 2A) [27, 28]. Upon closer inspection of the results and in particular when expanding the results using a more relaxed cut-off, many of the upregulated and down regulated genes are clustered together in the genome in putative metabolic pathway clusters. This becomes even more apparent when we look at all genes for which the cut-off for differential expression is 2log = 2. Several phosphate and iron related gene clusters were identified showing co-induction in high IA producing strains (Additional file 4).

Heterologous IA production in *A*. *niger* also results in the downregulation of many genes compared with the AB1.13 strain (Table 2B). In relation to organic acid production it is interesting to note that the expression of the *oahA* gene coding for oxaloacetate acetylhydrolase, the enzyme that hydrolyses oxaloacetate into oxalate and acetate, is downregulated in CitB#99. This observation is in line with our previous report were oxalic acid could not be detected in batch fermentations of CitB#99 [18]. Upon closer inspection also genes encoding malate synthase and isopropylmalate synthase are downregulated, assuming further rewiring of the organic acid pathway in our IA production hosts (Additional file 5). Remarkably among the downregulated genes are many that are involved with N transport and utilization. This could be caused by the use of NH_4_SO_4_, as sole N-source in IA production media, which could mediate nitrogen metabolite repression under high IA production conditions. In closer inspection of the selection of genes showing at least fourfold repression in IA overproducing strains besides the many N-source utilisation related functions (permeases etc.) also several secondary metabolite gene clusters were identified (Additional file 5).

Interestingly, in high IA producing conditions CitB#99 upregulates the expression of An07g00760 and An07g09220, when compared to AB1.13. This upregulation can already be observed in AB1.13 CAD strain that produces low titers of IA (Table 3) [20]. Genes An07g00760 and An07g09220 share sequence similarity with ATEG_06299 and ATEG_03709 that are identified in *A*. *terreus* as genes responsible for the biological degradation of IA [29] (Additional file 6). Sasikaran et al. [30] have also elucidated a similar pathway in pathogenic bacteria, suggesting a role in pathogenicity. As Chen et al. show the product of genes ATEG_06299 and ATEG_03709 are itaconyl-CoA transferase (IctA) and itaconyl-CoA hydratase (IchA) that together with citramalyl-CoA lyase (CclA) (ATEG_03186) degrade IA into the cellular building block chemicals pyruvate and acetyl-CoA. In *A*. *terreus* all three genes *ictA*, *ichA* and *cclA* are induced whereas in *A*. *niger* only *ictA* and *ichA* are induced. In contrast to what may have been expected for a catabolic pathway of a secondary metabolite as IA is, these IA bioconversion genes are not clustered in the genome of *A*. *niger*. More dedicated sequence analysis of the encoded proteins reveal that all three carry predicted mitochondrial targeting sequences, suggesting that IA conversion occurs in this compartment.

**Table 3:** Transcriptome data of genes involved in IA biosynthesis and bioconversion

Locus tag	Enzyme	Old locus tag	TargetP	AB 1.13 WT	AB 1.13 CAD	AB1.13 #49B	CitB#99
				RPKM	RPKM	RPKM	RPKM
	*IA biosynthesis cluster*						
	Major facilitator superfamily transporter (*mfsA*)		PM	0.30	2.36	222.56	721.88
	Cis-aconitate decarboxylase (*cadA*)		Cytosol	3.54	6887.96	3116.06	7417.64
	Mitochondrial tricarboxylite transporter (*mttA*)		Mito	0.19	0.54	477.02	1257.78
	*Citrate synthase*						
ANI_1_876084	Citrate synthase (*citA*)	An09g06680	Mito	284.82	269.02	255.71	238.09
ANI_1_1226134	Citrate synthase (*mcsA*)	An15g01920	Mito	76.60	61.89	82.55	85.78
ANI_1_1474074	Citrate synthase (*citB*)	An08g10920	Cytosol	3.05	3.10	2.87	10,838.65
ANI_1_2950014	Citrate synthase (*citC*)	An01g09940	Cytosol	463.10	370.88	438.08	96.44
	*PrpD family* (*CadA like*)						
ANI_1_1536084	Immune-responsive protein	An09g06220	Cytosol	0.30	0.23	0.36	0.43
ANI_1_2952014	Immune-responsive protein	An01g09950	Cytosol	552.06	456.98	383.21	165.41
ANI_1_2948014	2-Methylcitrate dehydratase	An01g09930	Cytosol	308.68	261.08	288.92	145.66
ANI_1_3352024	MmgE_PrpD superfamily protein	An02g14730	Cytosol	5.97	7.29	5.81	10.08
	*OahA class family*						
ANI_1_92174	Oxaloacetate acetylhydrolase (*oahA*)	An10g00820	Cytosol	1803.48	1122.69	323.44	9.81
ANI_1_2054064	Oxaloacetate acetylhydrolase	An07g08390	Cytosol	10.41	7.31	7.55	11.70
ANI_1_1800134	Oxaloacetate hydrolase class protein	An15g07720	Cytosol	49.63	70.43	95.09	67.85
	*IA bioconversion*						
ANI_1_1432064	Itaconyl-CoA transferase A (*ictA*)	An07g00760	Mito	13.07	108.35	170.34	375.89
ANI_1_676164	CoA transferase superfamily enzyme	An18g05120	Mito	11.10	9.36	11.79	9.99
ANI_1_2118064	Itaconyl-CoA hydratase A (*ichA*)	An07g09220	Mito	7.32	60.28	146.61	232.65
ANI_1_316154	HTD2 protein	An17g02190	Mito	23.69	22.26	22.39	26.75
ANI_1_1156014	Citramalate-CoA lyase (*cclA*)	An01g08610	Mito	10.17	12.59	21.43	19.54
ANI_1_864144	Trans-aconitate 2-methyltransferase (*tmtA*)	An16g06510	Mito	12.71	20.51	67.34	218.46
ANI_1_830134	UMTA methyltransferase family protein	An15g06160	Per/Cytosol	15.52	11.45	52.11	23.02

Interestingly, also another previously uncharacterized gene showed similar induction in expression as *ictA* and *ichA* in high producing IA strains. Upon closer inspection this gene product (An16g06510) shares 43% homology with *Escherichia coli* trans-aconitate 2-methyltransferase Tam, which is identified by Zhao et al. as potential gene product that esterifies itaconate into 1-methyl itaconate in the yeast *Saccharomyces cerevisiae* [31]. Tam reportedly shares the same molecular function as the yeast trans-aconitate 3-methyltransferase TMT1 i.e. methylation of spontaneously formed trans-aconitate in order to relieve cytosolic toxicity by trans-aconitate mediated inhibition of aconitase [32, 33].

### Deletion of *ictA* and *ichA*

In our transcriptome analysis we observed that the expression of *ictA* and *ichA* is upregulated in high IA producing strain CitB99. In *A*. *terreus* homologues of both gene products IctA and IchA have been shown to participate in a pathway that intracellularly convert IA into pyruvate and acetyl-CoA [29]. Based on these observations it was considered that knocking-out *ictA* and *ichA* would improve IA production. Bi-partite fragments were generated and transformed into CitB99. After transformation 89 colonies were visible on transformation plates. 24 colonies of each transformation were cultivated in microtiter plates for colony PCR. 4 colonies of CitB99 ΔICT were shown to be positive for deletion of the *ictA* gene and 6 colonies CitB99 ΔICH were positive for deletion of *ichA* (data not shown). To test for the effect of Δ*ictA* and Δ*ichA* on IA production in-time one CitB99 ΔICT and one CitB99 ΔICH strain were analyzed in flask experiments.

### Flask cultivation of CitB99 ΔICT and CitB99 ΔICH

**Fig. 1:**
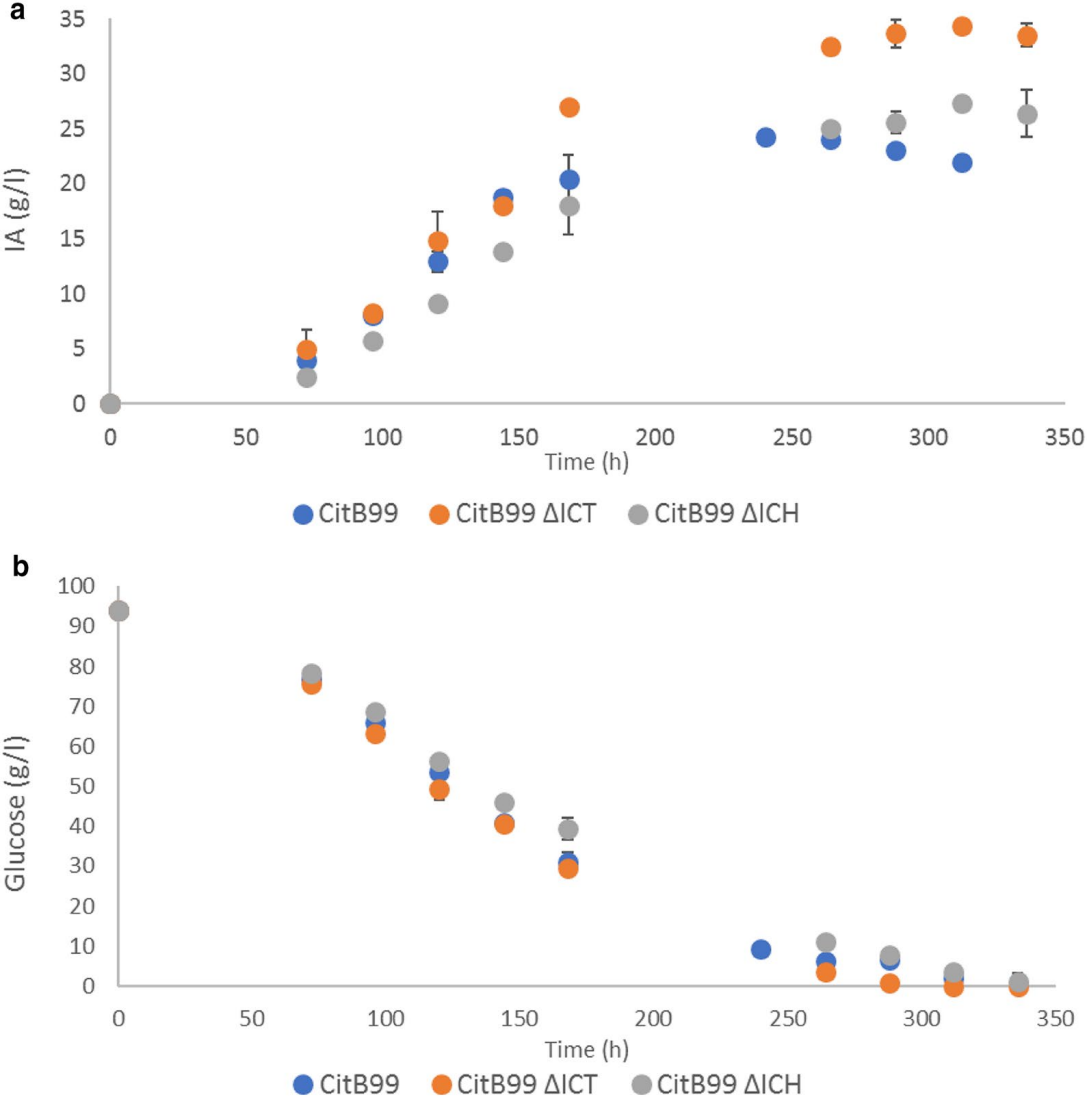
Non-shaking flask cultivation of CitB99 ΔICT, CitB99 ΔICH and CitB99. Non-baffled 500 mL shake flasks were inoculated with 1.0 × 10^6^ conidia/mL medium and incubated at 33 °C 0 RPM. The experiment was performed in duplicate. **a** Itaconic acid production, **b** glucose consumption during cultivation

The IA production of CitB99 ΔICT and CitB99 ΔICH were analyzed in flask experiments in order to test for the effect of Δ*ictA* and Δ*ichA* on IA production. In line with results obtained for CA production in *A*. *niger* [17] we have observed that under non-shaken conditions more reproducible CA and IA production levels could be obtained (Hossain AH et al. unpublished). Therefore Erlenmeyer flasks were inoculated with 1.0 × 10^6^ conidia/mL and incubated at 33 °C without shaking. From the results depicted in Fig. 1a it can be seen that IA production starts and proceeds very similar between CitB99 and CitB99 ΔICT up until 144 h of incubation, after which CitB99 ΔICT continues producing IA at a higher rate than CitB99. IA production in CitB99 reaches a plateau at 24.2 g/L after 240 h incubation before IA levels start to decline, due to degradation or bioconversion. Interestingly IA production in CitB99 ΔICT continues and reaches a final titer of 33.52 g/L IA after 336 h of incubation even after glucose is depleted in CitB99 ΔICT cultivation after 288 h (Fig. 1b). IA production in CitB99 ΔICH however, proceeds at a slower rate compared to CitB99 and CitB99 ΔICT. The final titer that is reached with this strain is much lower than CitB99 ΔICT at 26.39 g/L after 336 h but higher than the final titer of CitB99 due to the lack of degradation most likely. No side-product formation *e.g*. citrate or oxalate has been observed using HPLC analysis. These results show that IA detoxification can be inhibited by deleting *ictA* or *ichA* in *A*. *niger*. CitB99 ΔICT and CitB99 ΔICH were further tested in controlled-batch cultivations at 5-L scale.

### Controlled-batch cultivation

**Fig. 2:**
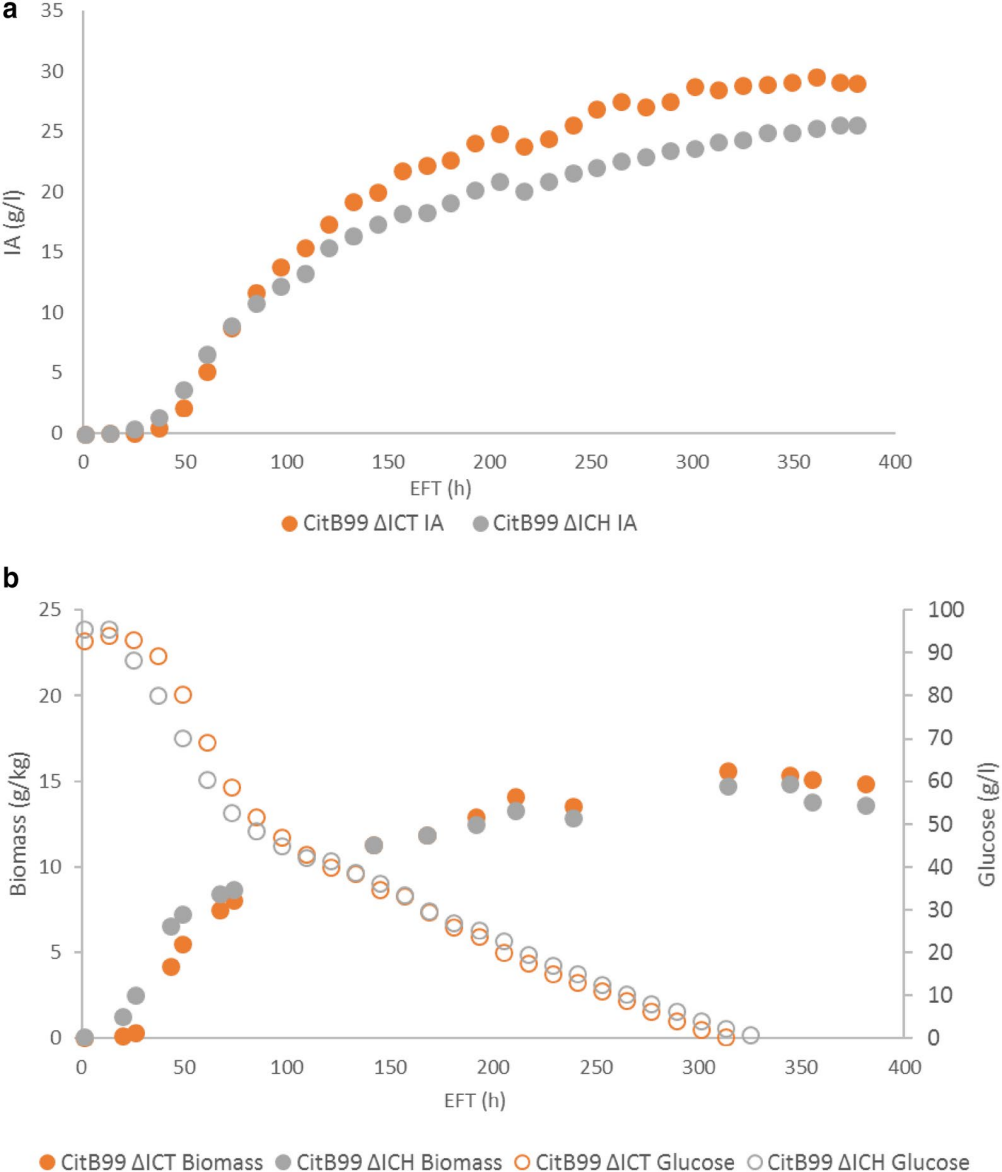
Controlled-batch cultivation of CitB99 ΔICT and CitB99 ΔICH. Three day grown pre-cultures were used to inoculate 5L controlled-batch cultivations. **a** IA production of CitB99 ΔICT and CitB99 ΔICH. **b** Glucose consumption and biomass formation of CitB99 ΔICT and CitB99 ΔICH

Previously, we have observed a slight decrease in IA titers during the end of controlled-batch fermentations [13, 18]. In order to further test the IA production of strains CitB99 ΔICT and CitB99 ΔICH we performed controlled-batch cultivations at 5L scale. The fermenters were inoculated with 100 mL 3 days old pre-cultures and DO was set at 20% saturation after inoculation. Glucose consumption, biomass formation and IA production started after 24 h of incubation. Glucose was depleted after 312 h for CitB99 ΔICT and after 324 h for CitB99 ΔICH at which point IA titer reached 28.7 and 24.3 g/L IA respectively (Fig. 2a). The fermentation was allowed to run for two more days to see if IA would drop in the glucose depleted cultures (Fig. 2b). The experiment was terminated after 380 h incubation in which no drop in IA levels were measured even after glucose had been depleted. The final titers reached were 29.2 g/L for CitB99 ΔICT and 25.7 g/L for CitB99 ΔICH.

### Deletion of *tmtA*

**Fig. 3:**
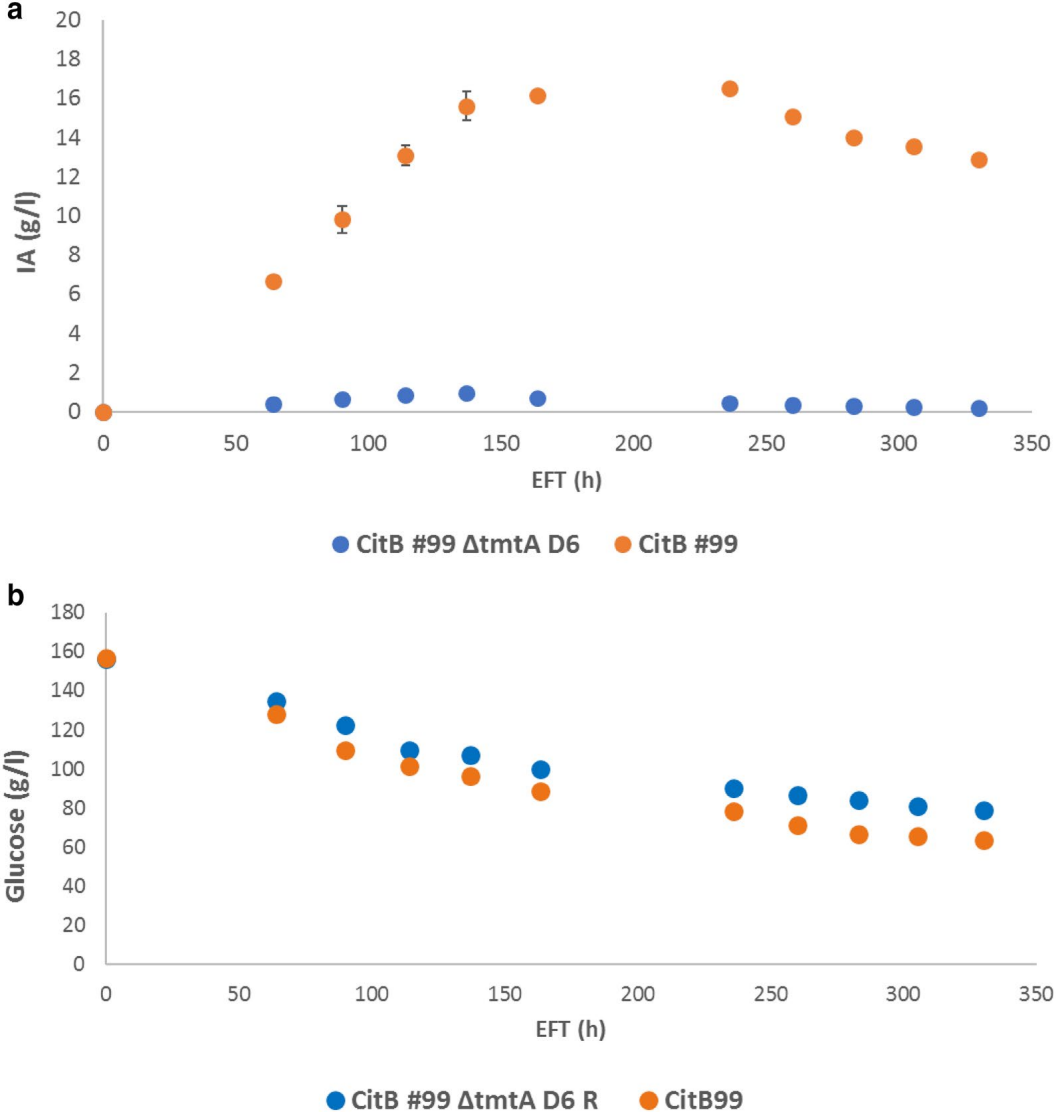
IA production of CitB99 Δ*tmtA* D6 and the parental CitB99 strain. Non-baffled 500 mL shake flasks were inoculated with 1.0 × 10^6^ conidia/mL medium and incubated at 33 °C and 250 RPM. **a** Itaconic acid production, **b** glucose consumption during cultivation

In order to investigate the role of TmtA in itaconate bioconversion, *tmtA* was disrupted in CitB99. After transformation 96 colonies were screened for knock-out using colony PCR and strain CitB99 Δ*tmtA* D6 was found to be a clean knock-out. IA production of CitB99 Δ*tmtA* D6 was further investigated by cultivation in shake flasks. However, HPLC analysis of shake flask cultivations with CitB99 Δ*tmtA* D6 and CitB99 (parental strain) in M12 ++ medium showed that IA production of CitB99 Δ*tmtA* D6 was negatively affected, achieving a max. titre of only 0.9 g/L IA compared to a max titre of 15.6 g/L IA achieved by the parental strain (Fig. 3a). Interestingly, glucose consumption was comparable between the two strains, suggesting that primary metabolism of glucose might not be affected in the Δ*tmtA* strain despite the low production of IA (Fig. 3b). Elevated levels of other organic compounds, including trans-aconitate and citrate, were not detected in the supernatant (data not shown). These results indicate that knock-out of *tmtA* does not result in the same desired phenotype in IA production as Δ*ictA* and Δ*ichA* has and as a result we did not proceed with 5L controlled-batch cultivations with this strain.

## Discussion

**Fig. 4:**
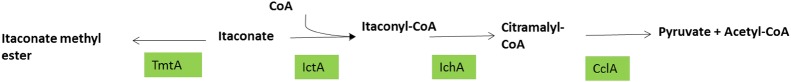
Hypothetical model of IA bioconversion in *A*. *niger*. Itaconate bioconversion presumably takes place in the mitochondrion where itaconate is converted to itaconyl-CoA by action of IctA and further hydrated to citramalyl-CoA by IchA. CclA facilitates the final conversion of citramalyl-CoA into pyruvate and acetyl-CoA. Concomitantly an parallel pathway can convert itaconate into 1-methyl itaconate

In our attempt at identifying unknown IA bioconversion pathways in *A*. *niger* we have performed a transcriptome analysis of high, medium and low IA producing strains. Interestingly, this analysis led to the identification of at least two novel organic acid bioconversion pathways that were not observed before (Fig. 4). IA is putatively converted into pyruvate and acetyl-CoA through the combined activity of IctA, IchA and CclA. Also, IA is putatively converted into 1-methyl itaconate through the activity of TmtA in yet another bioconversion pathway.

Our observation that knock-out strains of *ictA* and *ichA* show increased production of IA and are not able to degrade IA corroborate with the results of Chen et al. [29], that this pathway indeed converts IA intracellularly. Although CitB99 ΔICT strain is able to achieve higher titers than the CitB99 ΔICH strain, we observed that deletion of *ictA* appears to have the same effect as *ichA* i.e. both knock-out strains are unable to degrade IA. One remarkable feature of the parental strain CitB99 is that the strain performs variably in fermentations where high and low IA production can be seen (data not shown), whereas IA production appears to be more stable in CitB99 ΔICT (data not shown). Our results also suggest that no other enzyme is able to convert itaconate into itaconyl-CoA in the absence of IctA and also that itaconate bioconversion cannot proceed without the activity of IchA. Interestingly, CitB99 ΔICH achieves lower IA end titers than CitB99 ΔICT, possibly due to intracellular accumulation of itaconyl-CoA. However, the fate of IctA-mediated itaconyl-CoA accumulation in the CitB99 ΔICH strain remains unclear. Interestingly, the intracellular accumulation of itaconyl-CoA has been linked to decreased vitamin B12 levels in human brown adipocytes by Shen H et al. These researchers found that itaconyl-CoA can have a toxic influence by competitive inhibition of the mitochondrial vitamin B12-dependent methylmalonyl-CoA mutase (*mut*). This inhibition is mediated by converting vitamin B12 into the chemically inactive cob(II)alamin, thereby decreasing intracellular vitamin B12 levels [34]. Although fungi are not reported in literature to be able to synthesize nor use vitamin B12 as cofactor in biochemical reactions it cannot be excluded that a similar itaconyl-CoA mediated toxicity might be elicited [35]. Intracellular itaconyl-CoA accumulation might also exert a similar toxicity response as propionyl-CoA in *Aspergillus nidulans*. Brock M and Buckel W found that intracellular accumulation of propionyl-CoA mainly affects enzymes involved in glucose metabolism, thereby severely retarding growth [36]. However, as no apparent toxic effects of itaconyl-CoA accumulation were detected on growth and biomass formation in the CitB99 ΔICH strain, further research is required to investigate any potential toxic side-effects of itaconyl-CoA accumulation on fungal physiology.

Interestingly, the identified IA bioconversion pathway appears to share common features with bacterial C5-dicarboxylic acid metabolism, which can use C5 dicarboxylates such as itaconate, mesaconate and citramalate as growth substrates [37]. Pathogenic bacteria have been shown to use this pathway as a means to evade the hosts cellular defense mechanism during infection [30, 38]. Similarly *A*. *niger* may use this IA bioconversion pathway as defense mechanism during biological warfare. In nature *A*. *niger* and *A*. *terreus* share many common growth habitats and are constantly in conflict over scarce resources. However, the link between *Aspergillus niger* IA bioconversion and central metabolism is not known and is subject to further investigation.

Remarkably, in the high IA producing strain CitB#99 *ictA* and *ichA* show similar high levels of induction in expression compared to expression in the AB1.13 strain, however *cclA* does not show the same induction as *ictA* and *ichA*, suggesting that another protein has its function. An alternative possibility might be that IA is converted into an unknown compound in *A*. *niger*. We have detected unknown peaks in the HPLC samples of cultivations with high IA producing strains. The identification of these peaks and unraveling the link to IA detoxification however, is topic of ongoing research.

Interestingly, the second identified putative IA bioconversion pathway involves TmtA that supposedly converts IA into 1-methyl itaconate [31]. *tmtA* shows similar induction in expression as *ictA* and *ichA* from AB1.13 to CitB#99, possibly suggesting a role in IA bioconversion. *A*. *niger* TmtA shows sequence similarity of 28% with *S*. *cerevisiae* Tmt1 at a query coverage of 26% and 44% sequence similarity with *E*. *coli* Tam at 91% query coverage. Functional characterization of Tmt1 and Tam has led to the identification of trans-aconitate methyltransferase activity of both enzymes [32]. Remarkably, Tmt1 was also found to have low-level affinity for IA [33]. Katz et al. further elucidated that the major endogenous substrate for Tmt1 is an intermediate in the leucine biosynthesis pathway, 3-isopropylmalate, and that Tmt1 functions as a so called ‘moonlighting’ enzyme i.e. an enzyme that can perform multiple functions in different pathways [39]. The proposed role that Tmt1 plays in the citric acid cycle is detoxification of spontaneously formed trans-aconitate, an potent inhibitor of aconitase. However the physiologic function of methylating 3-isopropylmalate still requires elucidation [33, 39]. TmtA could perform a similar moonlighting function in *A*. *niger* where it methylates trans-aconitate and itaconate. Interestingly, deleting *tmtA* results in almost complete shutdown of IA production in *A*. *niger*. This may be caused by an accumulation of trans-aconitate in the mitochondrion and concomitantly the inhibition of aconitase, decreasing the flux to cis-aconitate. Interestingly, no other side-product such as CA was observed in the Δ*tmtA* strain. How TmtA exactly fits in heterologous IA production and overall central metabolism in *A*. *niger* is not entirely clear and is subject of further research. However from the data presented it is apparent that deletion of *tmtA* does not play any role in reducing IA bioconversion.

Remarkably, all of the enzymes involved in IA bioconversion IctA, IchA, CclA and TmtA carry predicted mitochondrial targeting sequences (Table 3). However, for IA bioconversion to take place in the mitochondrion, IA has to be first transported into the cytosol from the extracellular medium and concomitantly transported into the mitochondrion. It is currently unknown which transporters are involved in the transport across the plasma and mitochondrial membrane. However, in our transcriptome data multiple solute transporters have been identified making this a topic for further investigation.

In our research, apart from IA bioconversion pathways, we also looked at other potentially competing biosynthetic pathways that could hamper IA production. One possible way in which IA production might be hampered is when competing organic acid biosynthesis pathways are pulling precursor molecules, e.g. pyruvic acid or oxaloacetate, towards them. In *A*. *niger* one such competing pathway might lead to the formation of l-lactate [40]. Although a l-lactate dehydrogenase gene is annotated by sequence homology in *Aspergillus niger* (An04g08220) a functional l-lactate dehydrogenase enzyme has not been characterized yet [41]. Although *A*. *niger* strains are known to be potent oxalic acid producers, we have seen in our transcriptome data that the expression of the key gene for oxalate production i.e. *oahA* is significantly downregulated in CitB#99, indicating less competition from this pathway for precursor molecules.

In our research (Table 3), we have also found a non-canonical cytosolic citrate synthase similar to the previously identified *citB* [18]. This *citC* gene (An01g09940) is actually downregulated under *citB*-mediated improvement of IA production. Interestingly, also upon overexpression of *citC* we have observed similar positive effects on IA production (Additional file 7). Interestingly, similar as *citB* also *citC* appears to be clustered in a secondary metabolite gene cluster carrying two genes encoding CadA-like enzymes (An01g09930 and An01g09950). These results suggest that rewiring of secondary metabolism of *A*. *niger* towards IA production is much more intricate than we previously suspected.

Interestingly, our transcriptome data also indicates upregulation of phosphorus scavenging enzymes like acid phosphatase and phytase. Organic acid production in *A*. *niger* has been linked with phosphate depletion in the past [42, 43]. Recently, Upton et al. have shown that in phosphate limited citric acid production medium phosphate is quickly taken up by *A*. *niger* and stored as polyphosphate. The researchers further suggest that polyphosphate hydrolysis poses a constraint that limits growth and enables flux of carbon to organic acid production [44]. Whether a similar response occurs and if polyphosphate putatively fulfills a similar role during heterologous IA production in *A*. *niger* is not clear and warrants further research. Furthermore we have observed strong reduction in expression of genes encoding products that are involved in N transport and utilization, whose functional relation with heterologous IA production is not clear and also topic for further research.

## Conclusion

In our attempt to identify genes involved in putative IA bioconversion in *A*. *niger* we have analyzed a transcriptome dataset from batch fermentations of low, medium and high IA producing *A*. *niger* strains.

Transcriptome analysis has led us to two novel IA conversion pathways in *A*. *niger*. These pathways are not induced in non-IA producing conditions, strongly suggesting that they are specific for IA. One pathway shares homology with a recently identified IA degradation pathway identified in *A*. *terreus* through the combined action of IctA, IchA, and CclA. Upon deleting *ictA* or *ichA* we observed cessation of IA bioconversion and an increase in IA production titers.

Furthermore we identified a second putative IA bioconversion pathway in *A*. *niger* that supposedly converts IA into 1-methyl itaconate through the methylating activity of TmtA. Upon deleting *tmtA* we observed almost complete cessation of IA production, whereas overall growth and glucose consumption did not appear to be affected. Based on these observations we postulate that TmtA is an enzyme that esterifies spontaneously formed trans-aconitate and thereby relieves the cell of potential toxic effects of trans-aconitate accumulation. The results presented in this research contribute in further elucidation of heterologous IA production in *A*. *niger*.

## Additional files

**Additional file 1.** Schematic overview of the split-marker method.

**Additional file 2.** List of primers that were used to generate the split-markers for *ictA* and *ichA* deletion.

**Additional file 3.** Bioconversion of externally added IA to the cultivation medium. Strains AB1.13 CAD and CitB#99 were cultivated in IA supplemented medium (20 g/L) containing 0.1 and 0.5% glucose respectively. IA bioconversion was compared with medium containing 0.1% glucose.

**Additional file 4.** Transcriptome data of upregulated genes in CitB#99 vs AB1.13 with a 2logR cut off at 2.0.

**Additional file 5.** Transcriptome data of downregulated genes in CitB#99 vs AB1.13 with a 2logR cut off at -2.0.

**Additional file 6.** Protein BLAST alignments of IctA, IchA and CclA.

**Additional file 7.** IA production of *citC* overexpressing *A*. *niger* strain AB1.13 #49B-citC A12 Z.
