# Supplementary material for: Itaconic acid degradation in Aspergillus niger: the role of unexpected bioconversion pathways
Source: Fungal Biol Biotechnol. 2019 Jan 4;6:1. doi: 10.1186/s40694-018-0062-5 (PMC6320622; doi:10.1186/s40694-018-0062-5)
Supplement: Supplementary file 2 — Additional file 2. List of primers that were used to generate the split-markers for ictA and ichA deletion. [file 40694_2018_62_MOESM2_ESM.docx]

**List of primers used for generating split marker fragments and diagnostic PCR.**

| Nr | Construct | Primer name | Primer sequence 5’-3’ |
| --- | --- | --- | --- |
| 1 | 3-flank An07g09220/pyrE | T An07g09220-1 F | ACACGGCACAATTATCCATCGTTGGATAGGAGGTGAGCTG |
| 2 | 3-flank An07g09220/pyrE | T An07g09220-1,001 R | TGCGTTTCTTTTCCATGTCT |
| 3 | 3-flank An07g09220/pyrE | T An07g00760-8 F | ACACGGCACAATTATCCATCGCATGTTGGAGAGCATGGTT |
| 4 | 3-flank An07g09220/pyrE | T An07g00760-1,050 R | CTCGAATTAGCTCCAATTACC |
| 5 | 5-flank An07g09220/pyrE | P An07g09220-36 F | AGGCGTCAAAAATTCAAGTA |
| 6 | 5-flank An07g09220/pyrE | P An07g09220-1,500 R | CAATTCCAGCAGCGGCTTGGTTATTTTAATTGAGAGTGAA |
| 7 | 5-flank An07g00760/pyrE | P An07g00760-1,500 R | CAATTCCAGCAGCGGCTTGGTGCTGGTTGTGAATTGG |
| 8 | 5-flank An07g09220/pyrE | ppyrE-1f | AAGCCGCTGCTGGAATTGTTGTGTGTAGTTAGGCTAGATG |
| 9 | 3-flank An07g09220/pyrE | TpyrE-1,242 R | CAATTCCAGCAGCGGCTTAGTAGATGATTTGATTGCGAG |
| 10 | 3-flank An07g09220/pyrE | repeat An07g00760-1F | AAGCCGCTGCTGGAATTGGTAGACCTTATGCGACCG |
| 11 | 3-flank An07g09220/pyrE | repeat An07g09220-1 F | AAGCCGCTGCTGGAATTGGCCTACCTAAAATGTACAAG |
| 12 | 5-flank An07g00760/pyrE | P An07g00760-7 F | CAGCGGACTTCATGGACT |
| 13 | 5-flank An07g09220/pyrE | repeat An07g00760-500 R | CGATGGATAATTGTGCCGTGTGGTGCTGGTTGTGAATTG |
| 14 | 3-flank An07g09220/pyrE | repeat An07g09220-500 R | CGATGGATAATTGTGCCGTGTGGTTATTTTAATTGAGAGTGAA |
| 15 | 5-flank An07g09220/pyrE | PpyrE-1,545 R | CCAAGATCCCTCTGTGCA |
| 16 | 3-flank An07g09220/pyrE | TpyrE-1 F | ATCTCCGTACTTCTTCACCT |
| 17 | Start of *tmtA* promoter | 5-flank TmtA-4 F | GCTTTATCGATATT-TACCACCCA |
| 18 | *A. niger tmtA* locus,  upstream of *tmtA* gene | contr TmtA-31 F | AACCGGCGAAAGA-AAATGAA |
| 19 | *A. niger tmtA* locus,  downstream of *tmtA* gene | contr.TmtA-3,518 R | CGGAGCCCTATTC-AGTCTTAG |
| 20 | End of *tmtA* terminator | 3flank-TmtA-2,914 R | AGGGAAAGGAAG-GTGGTGA |
