# Supplementary material for: Itaconic acid degradation in Aspergillus niger: the role of unexpected bioconversion pathways
Source: Fungal Biol Biotechnol. 2019 Jan 4;6:1. doi: 10.1186/s40694-018-0062-5 (PMC6320622; doi:10.1186/s40694-018-0062-5)
Supplement: Supplementary file 6 — Additional file 6. Protein BLAST alignments of IctA, IchA and CclA. [file 40694_2018_62_MOESM6_ESM.docx]

BLASTP 2.8.0+

Reference: Stephen F. Altschul, Thomas L. Madden, Alejandro

A. Schaffer, Jinghui Zhang, Zheng Zhang, Webb Miller, and

David J. Lipman (1997), "Gapped BLAST and PSI-BLAST: a new

generation of protein database search programs", Nucleic

Acids Res. 25:3389-3402.

Reference for compositional score matrix adjustment: Stephen

F. Altschul, John C. Wootton, E. Michael Gertz, Richa

Agarwala, Aleksandr Morgulis, Alejandro A. Schaffer, and

Yi-Kuo Yu (2005) "Protein database searches using

compositionally adjusted substitution matrices", FEBS J.

272:5101-5109.

RID: ACYAKBCH014

Database: All non-redundant GenBank CDS

translations+PDB+SwissProt+PIR+PRF excluding environmental samples

from WGS projects

147,864,139 sequences; 54,185,348,171 total letters

Query= XP_001391168.1 CAIB/BAIF family enzyme IctA [Aspergillus niger CBS 513.88]

Length=417

Score E

Sequences producing significant alignments: (Bits) Value

XP_001215477.1 hypothetical protein ATEG_06299 [Aspergillus t... 706 0.0

ALIGNMENTS

>XP_001215477.1 hypothetical protein ATEG_06299 [Aspergillus terreus NIH2624]

EAU32843.1 hypothetical protein ATEG_06299 [Aspergillus terreus NIH2624]

Length=414

Score = 706 bits (1823), Expect = 0.0, Method: Compositional matrix adjust.

Identities = 343/415 (83%), Positives = 368/415 (89%), Gaps = 5/415 (1%)

Query 1 MPNTRPLVRAACHNL--SGMRHASTSATKKAGPLAGITVVSLEQAIAAPFCTRQLADLGA 58

M +RPL RA L S RH ST A K GPL GITVVSLEQAIAAPFCTRQLADLGA

Sbjct 1 MSLSRPLARAWAQTLAPSTRRHTSTQA-GKTGPLTGITVVSLEQAIAAPFCTRQLADLGA 59

Query 59 RVIKVERPGVGDFARNYDTRVNGLASHFVWTNRSKESLALDLKKPSDHSVLMRLLGRADV 118

RVIKVERPGVGDFARNYDTRVNGLASHFVWTNRSKESLALD+KKP DH VLMRLL +ADV

Sbjct 60 RVIKVERPGVGDFARNYDTRVNGLASHFVWTNRSKESLALDVKKPRDHQVLMRLLSKADV 119

Query 119 LVQNLAPGASARLGLSYDDLKAAHPSLIVCNISGYGPDGPYRDKKAYDLLIQSEAGMLSV 178

LVQNLAPGASARLGLS++DLKA +PSLIVCNISGYGPDGPYRDKKAYDLLIQSEAGMLSV

Sbjct 120 LVQNLAPGASARLGLSHEDLKATNPSLIVCNISGYGPDGPYRDKKAYDLLIQSEAGMLSV 179

Query 179 TGTGKEPAKVGISIADISAGSYAYSNILAALYQRERDPSKRGCNIDISMLESMVEWMGFP 238

TGTGKEPAKVGISIADISAG YAYSNILAAL QR++DP +RGCNIDISMLESMVEWMGFP

Sbjct 180 TGTGKEPAKVGISIADISAGCYAYSNILAALIQRDKDPKRRGCNIDISMLESMVEWMGFP 239

Query 239 MYYTYENAPGPTPAGASHAAIYPYGPFETGDGTVMLGIQNEREWAKFCDIVLGQPSLATN 298

MYYTY NAPGPTP GASHAAIYPYGPFETGDG+VMLGIQNEREW FCD VLG+P LAT+

Sbjct 240 MYYTYANAPGPTPTGASHAAIYPYGPFETGDGSVMLGIQNEREWTNFCDKVLGKPELATD 299

Query 299 ERFVNNSLRSQNRDELKKIICDVFSSLSAEQVIARLDAAAIANASVNDMQGVWNHPQLKA 358

RF NNSLRSQNR+ELK IIC+VFSSL+A+QVIARLD A+IANASVNDMQGVW HPQLKA

Sbjct 300 SRFANNSLRSQNREELKIIICEVFSSLTADQVIARLDGASIANASVNDMQGVWKHPQLKA 359

Query 359 RQRWTDVKTPAGSVPALLPPGMTMGDEDTYGARMDAVPDVGEHNKAILAELGLDE 413

R RWT+++TPAG+VPAL PPGM + ARMDAVP VGEHN++ILAELG+ E

Sbjct 360 RGRWTEIETPAGTVPALFPPGMDA--SANFAARMDAVPAVGEHNESILAELGMKE 412

Query= XP_001391996.1 hypothetical protein ANI_1_2118064 IchA [Aspergillus niger

CBS 513.88]

Length=361

Score E

Sequences producing significant alignments: (Bits) Value

XP_001212887.1 conserved hypothetical protein [Aspergillus te... 513 0.0

ALIGNMENTS

>XP_001212887.1 conserved hypothetical protein [Aspergillus terreus NIH2624]

EAU35511.1 conserved hypothetical protein [Aspergillus terreus NIH2624]

Length=364

Score = 513 bits (1322), Expect = 0.0, Method: Compositional matrix adjust.

Identities = 254/340 (75%), Positives = 284/340 (84%), Gaps = 4/340 (1%)

Query 26 RTFSIRPALRT--DTSASTIATSFLTRFQSLGPQTRSQTLDANQLQLLSLTLNRPSLFPN 83

R FS++ + R+ + +A ++A SFL+RFQS+GPQTRSQ LDANQLQLLSLTLNRPSL+PN

Sbjct 25 RRFSVQHSCRSAPEATAPSVAASFLSRFQSMGPQTRSQVLDANQLQLLSLTLNRPSLYPN 84

Query 84 SPSLSNTPTSLPTGTPLPAGYHLVYFTPAFLENELGADGTDTSYNPASPFTRRMWAGGEV 143

SPSLSN +PTGTPLP YHLVYFTPAFLE ELGADGTD SYNP PFTRRMWAGGEV

Sbjct 85 SPSLSNASGVVPTGTPLPPAYHLVYFTPAFLEGELGADGTDVSYNPEPPFTRRMWAGGEV 144

Query 144 HWPRGKDGKPNCLRVGQEVQETTRVLSAEPKVVRKTGEEMIVVGVEKEFRNENGVAVLDR 203

WPRG DGKPN LRVGQEVQETTRVLSAEPK++RKTG+EMIVV VEKEFRNE+GVAV+DR

Sbjct 145 QWPRGADGKPNPLRVGQEVQETTRVLSAEPKIIRKTGDEMIVVSVEKEFRNEHGVAVIDR 204

Query 204 RNWVFRKALTSPSPTSSSTPPATKAFNGPASSSTETSENVHTRTLRQTAVTLFRFSALTF 263

RNWVFRKAL S S++P + PAS ST T HTRTLRQTAVTLFRFSALTF

Sbjct 205 RNWVFRKALALASSPVSASPISVHLPALPASCSTSTVGKTHTRTLRQTAVTLFRFSALTF 264

Query 264 NPHKIHYSTPWARDVEGHKDIVVHGPLNLISILDLWRDTRKNGSGE-EVVLPEKISYRAT 322

NPHKIHYSTPWARDVEGHKDIVVHGPLNLISILDLWRDTR + + + ++LPE ISYRAT

Sbjct 265 NPHKIHYSTPWARDVEGHKDIVVHGPLNLISILDLWRDTRADSATDSSLLLPESISYRAT 324

Query 323 SPLYAEEEYRIVLEDGE-DGIGRVQIIAPGEVVAMKAEIQ 361

SPLYAEE YRIVL++ + DG+ RVQI P E VAMKAEI+

Sbjct 325 SPLYAEETYRIVLDEEQGDGVSRVQIFTPDEKVAMKAEIR 364

Query= XP_001389283.1 citrate lyase beta subunit CclA [Aspergillus niger CBS

513.88]

Length=323

Score E

Sequences producing significant alignments: (Bits) Value

XP_001212364.1 conserved hypothetical protein [Aspergillus te... 541 0.0

ALIGNMENTS

>XP_001212364.1 conserved hypothetical protein [Aspergillus terreus NIH2624]

EAU36460.1 conserved hypothetical protein [Aspergillus terreus NIH2624]

Length=322

Score = 541 bits (1393), Expect = 0.0, Method: Compositional matrix adjust.

Identities = 273/323 (85%), Positives = 299/323 (93%), Gaps = 1/323 (0%)

Query 1 MAARNTLRRALLYIPGSSQRFIDKSRTLTADCVAYDLEDSVTPHKKAEARSLVRRALDEP 60

MA+RNTLRRALLYIPGSSQRFIDKSRTLTADCVAYDLEDSVTPHKKAEARSLVRRALD+P

Sbjct 1 MASRNTLRRALLYIPGSSQRFIDKSRTLTADCVAYDLEDSVTPHKKAEARSLVRRALDQP 60

Query 61 APQGIRERAVRINSVDSGLALGDLTEVLKSPNLTTIVIPKVNTPSDLTFVNDVITHTLSQ 120

AP GI ERAVRINSVDSGLAL DLTEVL+SPNL+TIVIPKVN+ SDLTFV DVITHTLSQ

Sbjct 61 APTGILERAVRINSVDSGLALADLTEVLQSPNLSTIVIPKVNSASDLTFVTDVITHTLSQ 120

Query 121 QQQQDPSTPRPPISLLALVESAKSLTNLTQICASTPLLQGLIFAAEDFALDLSITRTPSL 180

+T RPPISLLALVESAKSLTNL+QICA++PLLQGLIFAAEDFALDLS+TRTP+L

Sbjct 121 LPPSQ-TTSRPPISLLALVESAKSLTNLSQICAASPLLQGLIFAAEDFALDLSLTRTPAL 179

Query 181 TEFLFARSMIATAARAANLPSTIDLVCTAYKSTKGDGSPPAVLEEECRDGRRLGFNGKQC 240

TEFLFARS IATAARAANLPSTIDLVCT YKS K DGSPPAVL++ECRDG+ LGFNGKQC

Sbjct 180 TEFLFARSAIATAARAANLPSTIDLVCTTYKSDKADGSPPAVLQQECRDGKNLGFNGKQC 239

Query 241 IHPSQVETAQAIFGPDPEEVKWAVRVCVADEKAARAGRGAWTLDGKMIDVPVAEKARAVV 300

IHPSQV T Q IFGP+ EEV+WAVRV +AD+KAA+AGRGAWTLDGKMID+PVAEKARA+V

Sbjct 240 IHPSQVSTVQQIFGPELEEVQWAVRVTIADDKAAKAGRGAWTLDGKMIDIPVAEKARAIV 299

Query 301 RKAEACGFDVGKLREEWGHQEPE 323

+KA+ACGF+V +LRE+W HQEPE

Sbjct 300 KKADACGFNVQELREKWQHQEPE 322
