# Supplementary material for: Itaconic acid degradation in Aspergillus niger: the role of unexpected bioconversion pathways
Source: Fungal Biol Biotechnol. 2019 Jan 4;6:1. doi: 10.1186/s40694-018-0062-5 (PMC6320622; doi:10.1186/s40694-018-0062-5)
Supplement: Supplementary file 5 [file 40694_2018_62_MOESM1_ESM.docx]

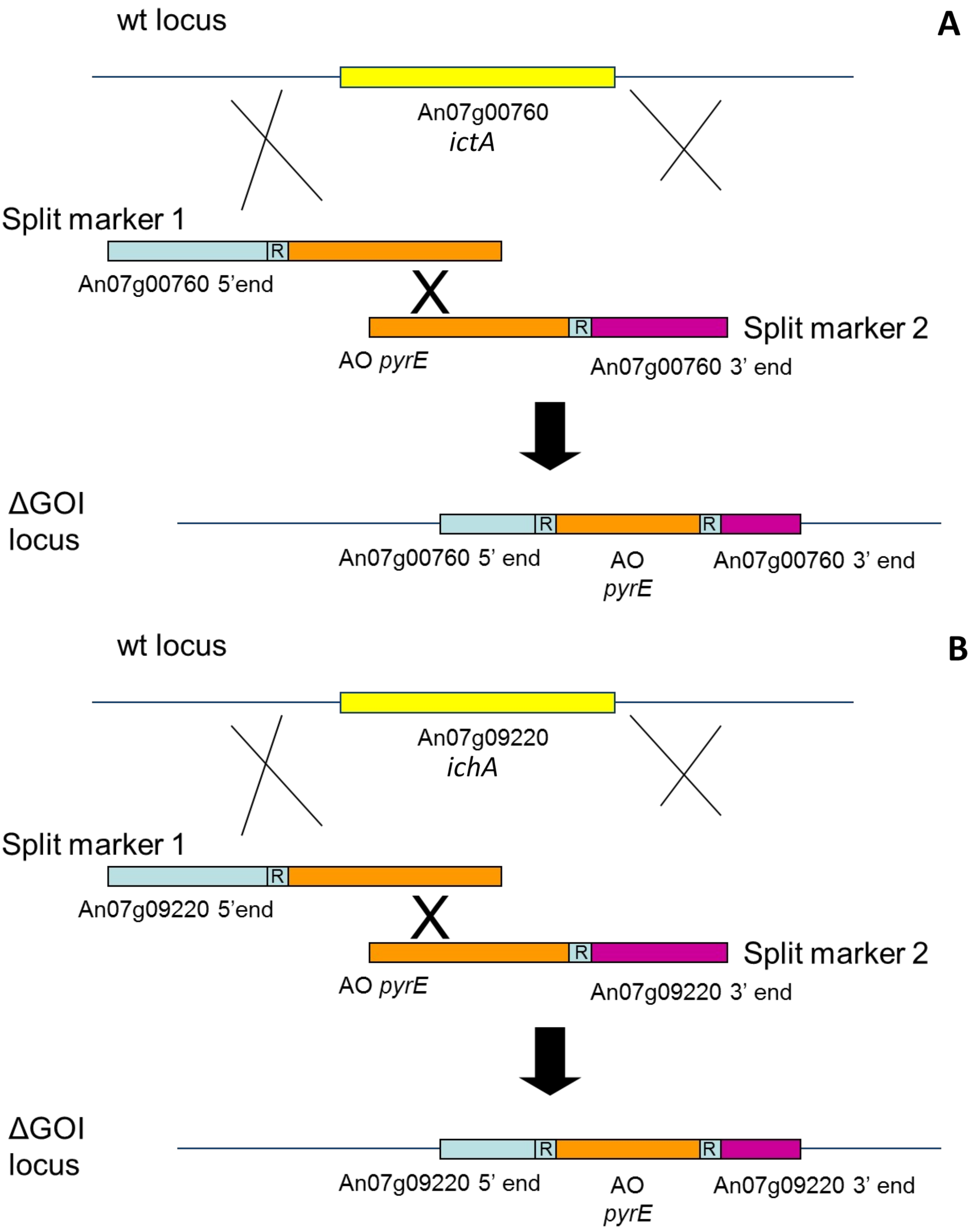
A
wt locus
An07g00760
ictA
Split marker 1
R
An07g00760 5’end
X
R
Split marker 2
AO pyrE
An07g00760 3’ end
ΔGOI locus
R
R
An07g00760 5’ end
AO pyrE
An07g00760 3’ end
B
wt locus
An07g09220
ichA
Split marker 1
R
An07g09220 5’end
X
R
Split marker 2
AO pyrE
An07g09220 3’ end
ΔGOI locus
R
R
An07g09220 5’ end
AO pyrE
An07g09220 3’ end
