# Supplementary material for: Itaconic acid degradation in Aspergillus niger: the role of unexpected bioconversion pathways
Source: Fungal Biol Biotechnol. 2019 Jan 4;6:1. doi: 10.1186/s40694-018-0062-5 (PMC6320622; doi:10.1186/s40694-018-0062-5)
Supplement: Supplementary file 6 [file 40694_2018_62_MOESM3_ESM.docx]

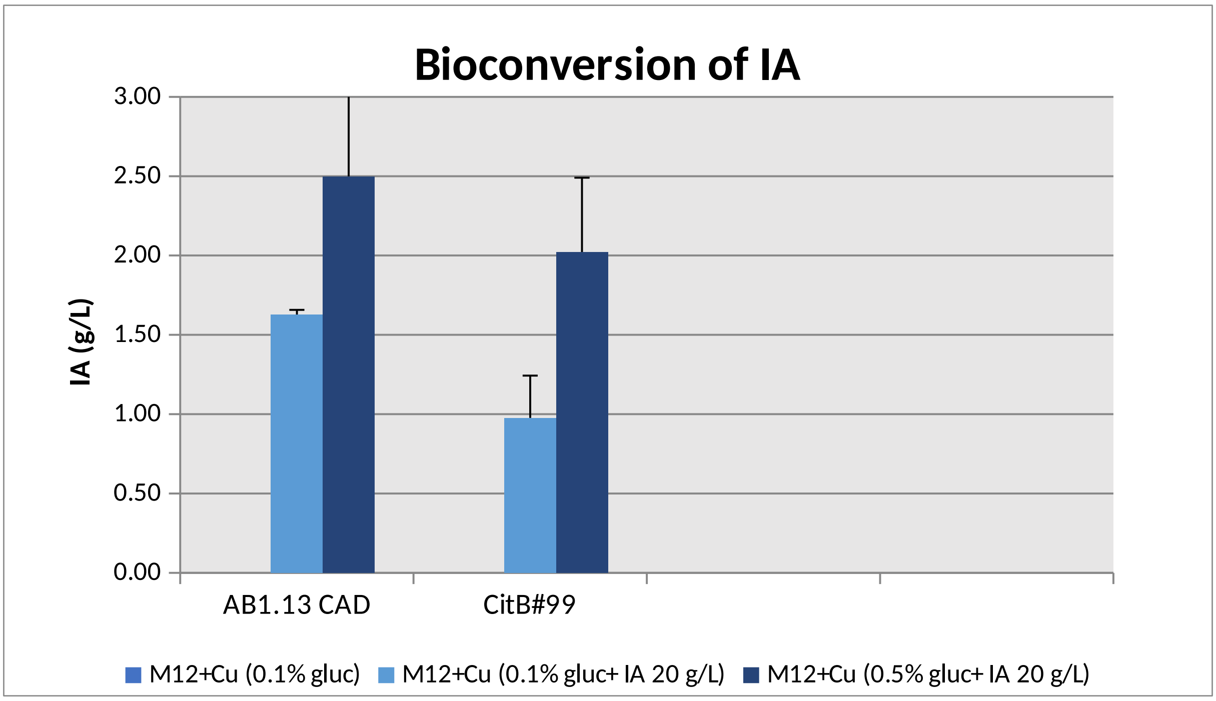
Bioconversion of IA
IA (g/L)
3.00
2.50
2.00
1.50
1.00
0.50
0.00
AB1.13 CAD
CitB#99
M12+Cu (0.1% gluc)
M12+Cu (0.1% gluc+ IA 20 g/L)
M12+Cu (0.5% gluc+ IA 20 g/L)
