# Supplementary material for: Itaconic acid degradation in Aspergillus niger: the role of unexpected bioconversion pathways
Source: Fungal Biol Biotechnol. 2019 Jan 4;6:1. doi: 10.1186/s40694-018-0062-5 (PMC6320622; doi:10.1186/s40694-018-0062-5)
Supplement: Supplementary file 7 [file 40694_2018_62_MOESM7_ESM.docx]

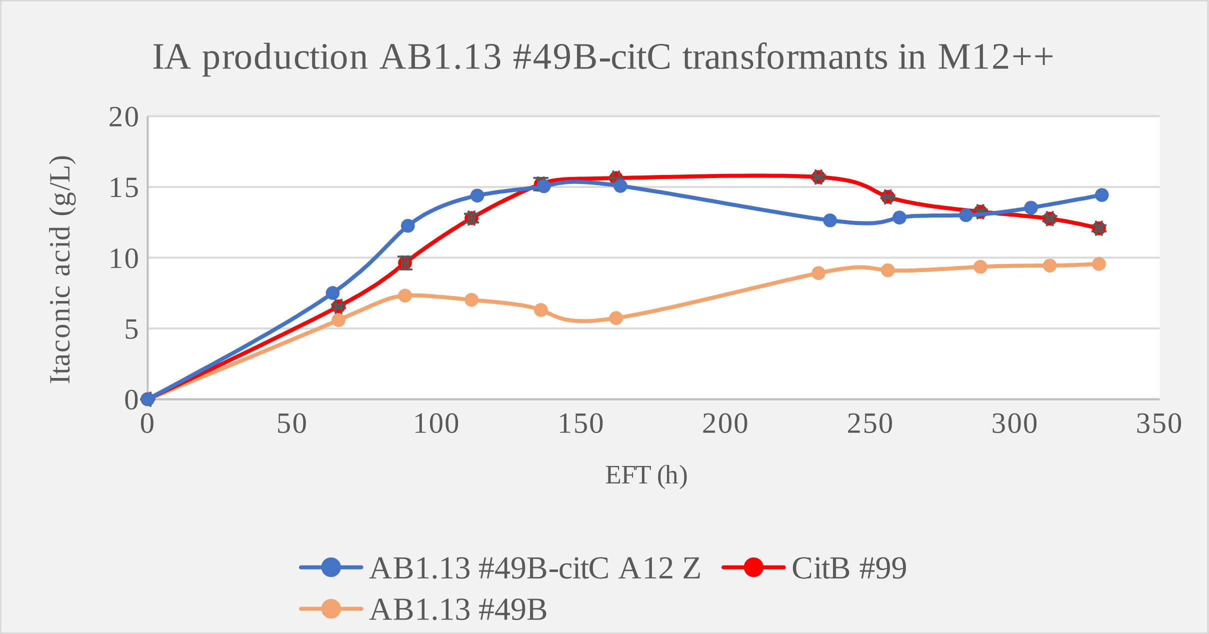
IA production AB1.13 #49B-citC transformants in M12++
Itaconic acid (g/L)
0
5
10
15
20
0
50
100
150
200
250
300
350
EFT (h)
AB1.13 #49B-citC A12 Z
CitB #99
AB1.13 #49B
